# Effect of Exercise Training on Fat Loss—Energetic Perspectives and the Role of Improved Adipose Tissue Function and Body Fat Distribution

**DOI:** 10.3389/fphys.2021.737709

**Published:** 2021-09-24

**Authors:** Kristoffer Jensen Kolnes, Maria Houborg Petersen, Teodor Lien-Iversen, Kurt Højlund, Jørgen Jensen

**Affiliations:** ^1^Steno Diabetes Center Odense, Odense University Hospital, Odense, Denmark; ^2^Department of Internal Medicine, Randers Regional Hospital, Randers, Denmark; ^3^Department of Physical Performance, Norwegian School of Sport Sciences, Oslo, Norway

**Keywords:** exercise, high intensity interval aerobic training, fat loss, adipose tissue function, inflammation, type 2 diabetes, obesity

## Abstract

In obesity, excessive abdominal fat, especially the accumulation of visceral adipose tissue (VAT), increases the risk of metabolic disorders, such as type 2 diabetes mellitus (T2DM), cardiovascular disease, and non-alcoholic fatty liver disease. Excessive abdominal fat is associated with adipose tissue dysfunction, leading to systemic low-grade inflammation, fat overflow, ectopic lipid deposition, and reduced insulin sensitivity. Physical activity is recommended for primary prevention and treatment of obesity, T2DM, and related disorders. Achieving a stable reduction in body weight with exercise training alone has not shown promising effects on a population level. Because fat has a high energy content, a large amount of exercise training is required to achieve weight loss. However, even when there is no weight loss, exercise training is an effective method of improving body composition (increased muscle mass and reduced fat) as well as increasing insulin sensitivity and cardiorespiratory fitness. Compared with traditional low-to-moderate-intensity continuous endurance training, high-intensity interval training (HIIT) and sprint interval training (SIT) are more time-efficient as exercise regimens and produce comparable results in reducing total fat mass, as well as improving cardiorespiratory fitness and insulin sensitivity. During high-intensity exercise, carbohydrates are the main source of energy, whereas, with low-intensity exercise, fat becomes the predominant energy source. These observations imply that HIIT and SIT can reduce fat mass during bouts of exercise despite being associated with lower levels of fat oxidation. In this review, we explore the effects of different types of exercise training on energy expenditure and substrate oxidation during physical activity, and discuss the potential effects of exercise training on adipose tissue function and body fat distribution.

## Introduction

Obesity, and in particular abdominal obesity, increases the risk of several diseases, including type 2 diabetes mellitus (T2DM), cardiovascular disease (CVD), non-alcoholic fatty liver disease (NAFLD), polycystic ovarian syndrome (PCOS), severe COVID-19 disease, and certain types of cancer (Kopelman, 2000; Stefan et al., 2021). The prevalence of obesity worldwide has increased dramatically over the last few decades (Forouhi and Wareham, 2014). Increased energy intake and insufficient physical activity result in a positive energy balance, the main cause of weight gain that subsequently leads to obesity. Despite its simple equation, energy intake vs. output, obese people find it difficult to maintain a negative energy balance over time, highlighting the need to find methods that can achieve a better metabolic outcome.

Lifestyle adaptation is the primary intervention for managing obesity and its related diseases (Wadden et al., 2012). Weight loss can be achieved by increasing energy expenditure and/or reducing calorie intake. Energy expenditure during physical activity depends on the type of exercise as well as its intensity and duration. Theoretically, the energy content in 1 kg of fat allows a person weighing 70 kg to run approximately 125 km (Frayn, 1983; Litleskare et al., 2020). In reality, however, humans need to run much further to oxidize 1 kg of fat because carbohydrate oxidation contributes to energy expenditure. Protein oxidation is determined by protein intake, while amino acid oxidation has a minimal effect on total energy expenditure during exercise, and is therefore not included in the calculations in this review (Tarnopolsky, 2004).

Indirect calorimetry is used not only to measure cardiorespiratory fitness (*V*O_2*max*_) but also to provide estimates of the energy expenditure and whether the substrate utilized is derived from fat or carbohydrate oxidation (Frayn, 1983). During low-to-moderate intensity exercise (30–65% of *V*O_2*max*_), fat is the major source of energy, and this type of exercise can be maintained for several hours. In contrast, carbohydrates become the principal energy source during high-intensity exercise (>85% of *V*O_2*max*_), which can rarely be maintained for more than half an hour, except by elite endurance athletes (Romijn et al., 1993). Additionally, a very low rate of fat oxidation is observed during high-intensity exercise (Romijn et al., 1993; Andersson Hall et al., 2016). These observations underlie the belief that exercise training with low-to-moderate intensity and of long duration is the best way to lose fat mass. However, high-intensity short-duration exercise training has become increasingly popular due to its time-related effectiveness (Keating et al., 2017).

High-intensity interval training (HIIT) and sprint interval training (SIT) are among the most popular and most studied high-intensity training regimens. Here, we use the nomenclature described in the review by MacInnis and Gibala (2017). HIIT is defined as a near-maximal effort, often performed as bouts of 2–6 min of work at 85–95% of maximal heart rate (MHR) with 2–3 min of rest between bouts. SIT is defined as a maximal or supramaximal effort and is often performed as all-out bouts of 30 s or less, with 2–5 min of rest between bouts. Moderate-intensity continuous training (MICT) consists of continuous exercise at lower intensities. Regarding the ability of the different exercise regimens to reduce visceral adipose tissue (VAT), HbA1c, and fasting glucose, HIIT and SIT seem to have effects that are at least similar to those of MICT in both healthy and diabetic subjects (Burgomaster et al., 2008; Fealy et al., 2018; Søgaard et al., 2018; Winding et al., 2018; Sabag et al., 2020). HIIT and SIT also efficiently reduce total fat mass, despite carbohydrates being the predominant source of energy during the exercise bouts (Trapp et al., 2008; Kuo and Harris, 2016). As the amount of fat lost after a relatively long period of HIIT and SIT is disproportionately larger than the estimated utilization within the HIIT or SIT sessions, the mechanisms explaining this fat-reducing effect are of interest (Kuo and Harris, 2016).

Fat is predominantly stored in adipocytes in various depots throughout the body. Fat localization is a strong predictor of T2DM, NAFLD, and CVD (Mooney et al., 2013). Abdominal obesity, especially increased visceral fat, is associated with an increased risk of the above-mentioned diseases (Karpe and Pinnick, 2015), rendering it an important target in attempts to improve metabolic health. In addition, obesity-related insulin resistance is associated with impaired insulin-mediated inhibition of lipolysis in adipose tissue (AT), resulting in an increased efflux of free fatty acids (FFAs) into the blood and, consequently, ectopic lipid deposition (Snel et al., 2012). Impaired insulin-mediated inhibition of lipolysis is further exacerbated by AT dysfunction (Crewe et al., 2017). Evidence suggests that dysfunctional AT promotes systemic low-grade inflammation, fat overflow, and, hence, ectopic lipid deposition, which further contributes to insulin resistance (Virtue and Vidal-Puig, 2010). Although AT dysfunction is improved by weight loss, the specific impact of exercise training on the former is still unclear (Murphy et al., 2017). Nevertheless, it is thought that the exercise-mediated improvement in metabolic health—including increased insulin sensitivity—may involve improved AT function, comprising an increased ability to store and oxidize fat, reduced fat overflow, and decreased systemic low-grade inflammation (Park et al., 2014). This may drive a reduction in abdominal fat levels, including VAT, and reduce ectopic lipid deposition in the liver and skeletal muscle, as well as in other organs.

Here, we review the effect of exercise training on body fat, focusing on energy expenditure and fat metabolism. The mechanisms explaining the benefits of high-intensity exercise training on AT function and metabolic flexibility, despite minimal β-oxidation during the training sessions, are also discussed.

## Energy Expenditure in the Human Body

During evolution, movement was necessary for humans to obtain food for survival. Although movement to obtain food and flee from predators remains a prerequisite for animals living in the wild, this is no longer true for most humans, most of whom have an increasingly sedentary lifestyle. Most people exercise to maintain fitness and prevent obesity rather than moving to hunt and gather food (Jensen and O’Rahilly, 2017). Furthermore, pre-prepared food is cheap, energy-dense, and abundant. Humans easily eat more calories than they can expend (O’Rahilly, 2016). As we are still adapted for a life as hunter–gatherers, these significant lifestyle changes have resulted in an increasing prevalence of obesity.

To create a framework for understanding energy expenditure, we introduce and briefly describe some relevant terms. Total daily energy expenditure (TDEE) is the total amount of energy used in a day and is measured using the doubly labeled water (DLW) method (Westerterp, 2017). TDEE depends on resting energy expenditure (REE) and activity-related energy expenditure (AEE). REE is the energy used during complete rest and AEE refers to the energy used during daily activity and/or exercise. AEE and REE are usually measured by indirect calorimetry.

Precise calculations of energy intake require carefully controlled experimental conditions. The correct collection of such data is difficult in population studies as people normally underreport food intake and overestimate activity when questionnaires are used to address energy balance (Fogelholm et al., 2006; Stubbs et al., 2014). TDEE depends on the REE, free-living activities, and the amount of physical activity performed, collectively called AEE. A fraction (∼10%) of the TDEE can be explained by the thermic effect of food (TEF) (Westerterp, 2004). Even though cardiorespiratory fitness is a predictor of the TEF, the effect of exercise on the TEF remains uncertain (Calcagno et al., 2019). Accordingly, the TEF will not be included in the calculations in this review. The gold standard for measuring total energy expenditure is the DLW method (Westerterp, 2017). This technique is expensive and time-consuming, and it takes 1–3 weeks to collect good data. Energy expenditure can also be measured by direct calorimetry, which measures the amount of energy lost as heat from the body; or by indirect calorimetry, which measures oxygen utilization and carbon dioxide production (Ndahimana and Kim, 2017). Because indirect calorimetry is a standard procedure in exercise physiology laboratories, we focus on this method in this review.

Indirect calorimetry measures the volume of oxygen (O_2_) and carbon dioxide (CO_2_) inspired and expired. The synthesis of adenosine triphosphate (ATP) in mitochondria requires oxygen to capture electrons from the electron transport chain during the oxidation of carbohydrates and fat. The coupling between oxygen uptake (*V*O_2_) and CO_2_ production (*V*CO_2_) has known stoichiometry, which allows the calculation of substrate oxidation and energy expenditure from *V*O_2_ and *V*CO_2_ measurements obtained *via* indirect calorimetry. Carbohydrate and fat metabolism is also well characterized (Frayn, 1983). The oxidation of 1 g of glucose requires 0.747 L of O_2_ and provides 17 kJ (4 kcal), that of 1 g of fat requires 2.03 L of O_2_ and provides 37 kJ (9 kcal), and that of 1 g of protein 0.966 L of O_2_ and provides 17 kJ (4 kcal). Knowing these values allows for the calculation of energy expenditure from oxygen consumption and the respiratory exchange ratio (RER). The RER further provides information regarding whether the source of the energy utilized originates from carbohydrates or fat. The oxygen consumption determines the amount of substrate oxidized and, therefore, the energy released within the body.

The REE is the amount of energy a person uses at rest and is related to body size and composition (Henry, 2005). On a population basis, the REE accounts for ∼60% of the total energy expenditure. The average oxygen consumption for an adult human sitting at rest is approximately 3.5 mL/(kg⋅min^–1^) (Henry, 2005), which corresponds to approximately 1,750 kcal for a person weighing 70 kg sitting still all day (Jetté et al., 1990). On a population basis, physical activity rarely accounts for more than 40% of the total energy expenditure (Westerterp, 2000). Unless a person is very active or a professional athlete, it is very difficult for anyone to exceed their REE during activity.

## Energy Expenditure During Exercise

The AEE during physical activity depends on both daily activities and exercise. Energy expenditure during a session of endurance exercise depends on the duration, type, and intensity of the exercise, as well as cardiorespiratory fitness. The maximal oxygen uptake (*V*O_2*max*_) is a reflection of cardiorespiratory fitness and is indicative of how much energy a person can utilize during 1 min. *V*O_2*max*_ is usually related to body weight. In some instances, however, relating *V*O_2*max*_ to fat-free mass is of more value as body composition can differ substantially within the same body mass index (BMI) range.

Members of our group often examine well-trained, lean young males with a *V*O_2*max*_ of 50–75 mL/(kg⋅min^–1^) (Rustad et al., 2016; Sollie et al., 2018); in contrast, the *V*O_2*max*_ for untrained middle-aged males is approximately 45 mL/(kg⋅min^–1^) (Langleite et al., 2016; Jelstad et al., 2019). Obese people have reduced cardiorespiratory fitness and their *V*O_2*max*_ may be reduced to 20 mL/(kg⋅min^–1^) or less (Christ-Roberts et al., 2004; Vind et al., 2011); however, this is not necessarily solely due to low cardiorespiratory fitness but may also result from an increase in body weight. Furthermore, obese people may experience decreased mobility. A combination of low cardiorespiratory fitness and reduced mobility helps explain why exercise becomes an overwhelming task for an obese person, especially knowing that they must run 100 km to get rid of 1 kg of fat (see BOX 1).

**Box 1. Energy expenditure calculation during running.**

Energy expenditure during treadmill running is often estimated by oxygen uptake. External work (the force causing displacement) is difficult to measure in a person running. Instead, running economy (RE) is calculated according to an oxygen consumption of ∼0.2 L/(kg⋅km^–1^), equal to ∼1 kcal/(kg⋅km^–1^) (Litleskare et al., 2020). Therefore, it costs a person weighing 77 kg approximately 7,700 kcal to run 100 km, corresponding to the energy in 1 kg of fat. (Formula: RE = VO_2_⋅kg^–1^⋅km^–1^).

**FIGURE 1:**
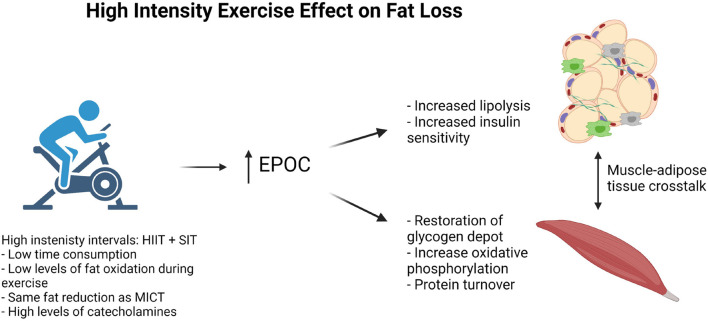
High intensity exercise effect on fat loss.

Oxygen uptake increases gradually during high-intensity exercise, leading to an O_2_ deficit (Krogh and Lindhard, 1920). Moreover, a large O_2_ deficit develops during exercise performed at above the anaerobic threshold (Medbø and Tabata, 1989). This makes it difficult to use indirect calorimetry to calculate energy expenditure during HIIT and, especially, SIT. Generally, the oxygen debt acquired during anaerobic activity will be an underestimate of the energy expenditure during the anaerobic work. Combining this O_2_ deficit with the excess post-exercise oxygen consumption (EPOC) effect, continuous measurement with indirect calorimetry in the recovery period is required for the estimation of extra oxygen consumption used to pay for the oxygen debt and EPOC-related processes (Figure 1).

Total work in healthy young males during cycling SIT with four intervals of 30 s corresponds to an energy use of ∼75 kcal (McCartney et al., 1986; Spriet et al., 1989), whereas during a session of MICT, such as running for 45 min at 75% MHR, a young healthy person weighing 70 kg uses 700 kcal. This clearly indicates that SIT requires only a fraction of the energy expended in MICT. As previously mentioned, HIIT is often performed as 4–7 bouts of work at 85–95% of MHR with 2–3 min of rest between bouts (Bækkerud et al., 2016; Granata et al., 2016; Meinild Lundby et al., 2018). Depending on the length and number of bouts, energy expenditure during HIIT is often the same, or similar to, that for MICT of the same duration (MacInnis and Gibala, 2017).

## Energy After Exercise

Although fat loss is the same, the workload in MICT is much greater than that in HIIT and SIT, indicating that fat loss during HIIT and SIT is greater than expected. Several mechanisms can help explain this surprising outcome. First, as has been suggested, REE may increase in response to high-intensity training; however, no direct evidence exists for such an effect (Karstoft et al., 2017). Secondly, fat loss could result from participants reducing their relative food intake in response to HIIT during a particular study. However, there is evidence to suggest this is not the case (Rosenkilde et al., 2013). Thirdly, although exercise-induced *V*O_2_ utilization drops immediately after cessation of exercise, it remains elevated for up to 24 h after an exercise bout compared with that in the resting state (Tucker et al., 2016). This increase in *V*O_2_ utilization is known as EPOC. There is evidence that the duration of an exercise bout shows a linear relationship with EPOC, whereas an exponential relationship exists between increasing exercise intensity and EPOC (Moniz et al., 2020). Despite these findings, results regarding whether EPOC is responsible for the fat loss associated with high-intensity exercise are conflicting (Tucker et al., 2016).

Biological processes responsible for an increase in EPOC include enhanced protein synthesis related to muscle remodeling and the restoration of intracellular lipid and glycogen deposits in both the liver and skeletal muscle (Moniz et al., 2020) (Figure 1). As energy stores are limited in skeletal muscle after exercise, energy must be supplemented from other tissues, including AT. Thus, AT must adapt to the increased energy demands of the skeletal muscle. We hypothesize that, among these adaptations, there is an improvement in AT function that leads to a more favorable shift between carbohydrate and fat oxidation, called metabolic flexibility (Goodpaster and Sparks, 2017). Metabolic flexibility is the ability to shift from fat to carbohydrate oxidation with, for instance, increasing exercise intensity, and then back from carbohydrate to fat oxidation during rest. Improved metabolic flexibility results in a larger fraction of total energy expenditure coming from β-oxidation in the resting state, thereby increasing total fat oxidation. Furthermore, an increase in mitochondrial oxidative phosphorylation in adipocytes could contribute to an increase in EPOC. However, evidence that this occurs in adult humans in response to exercise is scarce, and will not be further discussed (Cannon and Nedergaard, 2011).

## Fat and Excess Adiposity

In the healthy state, most fat is stored in AT. There are two types of AT, namely, white and brown AT. In infants, brown AT is involved in thermogenesis, while its role in adult humans is still debated and has been reviewed elsewhere (Rosen and Spiegelman, 2014). White AT is specialized for fat storage and is distributed around different depots in the body. Apart from storing energy, white AT is also a multifunctional organ with endocrine functions that affect whole-body metabolism (Scheja and Heeren, 2019). The energy stored in AT allows humans to survive for long periods without food. Young healthy people with ∼20% body fat can survive for 1–2 months without food (Cahill, 1970). The longest documented period of starvation in a human is 382 days (Stewart and Fleming, 1973). During this period, the weight of the individual declined from 207 to 82 kg, corresponding to a weight loss of 125 kg. Assuming (although fictive, as fat loss only is unrealistic in such a long fast) that all this weight loss was related to fat, with an energy content of 7,700 kcal/kg, this equates to ∼1,000,000 kcal, i.e., an average of ∼2,500 kcal/day during the 382 days of fasting (Stewart and Fleming, 1973).

High body fat content not only causes major health problems but also prevents people from exercising effectively. Total fat mass is most accurately measured by magnetic resonance imaging (MRI) or computed tomography (CT) (Wells and Fewtrell, 2006). However, MRI and CT are time-consuming and expensive, and cheaper and faster measurement methods such DXA scan and bioimpedance, or anthropometric measurements such as BMI or waist–hip ratio, are often used instead to evaluate body composition and metabolic health. BMI only reflects weight in relation to height and is, therefore, a suboptimal measurement as humans can attain similar BMIs with different body compositions. However, on a population level, an increase either in BMI or total fat mass can increase the risk of obesity-related diseases (Van Pelt et al., 2002; Bays et al., 2007).

## Metabolic Differences in Fat Depots

From a metabolic perspective, it is important to distinguish between different fat depots within the body. White AT is divided into two main types—VAT and subcutaneous white adipose tissue (SAT). VAT is fat located in different depots surrounding internal organs, whereas SAT is located under the skin throughout most of the body. In obesity, most SAT is located on the abdomen or hips. An excess of abdominal fat, termed android obesity, is more common in men, whereas excessive fat storage on the hips and legs (glutofemoral fat mass), often referred to as gynoid obesity, is more common in women. Greater amounts of abdominal (android) fat increase the risk of T2DM, NAFLD, and CVD, whereas peripheral fat centered at the hips and legs (gynoid) has been suggested to exert a protective effect against CVD (Mooney et al., 2013; Karpe and Pinnick, 2015). Studies have shown that increased gynoid obesity is associated with more favorable lipid and glucose metabolism independently of the amount of VAT (Stefan, 2020).

Increasing the amount of VAT alone can increase the risk of the above-mentioned diseases independently of BMI and total fat mass, underlining the importance of fat localization (Tchernof and Després, 2013). The exact mechanism underlying why VAT is more harmful than SAT remains unknown. Features that could make VAT more metabolically harmful include differences in innervation and that VAT has direct access to the portal vein, leading to the released fat finding its way directly to the liver (Nguyen et al., 2014). Furthermore, adrenergic receptors expressed in adipocytes in VAT differ from those expressed in SAT adipocytes (Stefan, 2020). It has also been speculated that fat is stored in the visceral depots when the fat-storing capacity in the subcutaneous depots is exceeded, indicating an overflow of fat and ectopic lipid deposition (Snel et al., 2012). Regardless of the mechanisms, reducing the amount of abdominal visceral fat is of great interest from a health perspective.

## Adipose Tissue and Its Function

Fat has an energy content of 9,000 kcal/kg. However, because AT in humans also contains a small amount of water and other cells, the actual *in vivo* energy content in 1 kg of AT is approximately 7,700 kcal (Flatt, 1995; Heymsfield et al., 2012). Despite accounting for only 50–60% of the total cell number, adipocytes comprise over 85% of the volume in white AT (Lenz et al., 2020). In adipocytes, fat is stored as triacylglycerol in lipid droplets that make up > 95% of the intracellular content (Arner, 2005). That almost all the volume of AT is comprised of fat is indicative of its importance as a storage depot. However, even though the volume of non-adipocytes in AT is limited, these cells exert a significant influence on AT function. Non-adipocytes in AT include immune cells, preadipocytes, endothelial cells, and fibroblasts (Lenz et al., 2020), known collectively as the stromal vascular fraction (Bourin et al., 2013). These cells are responsible for adipogenesis, angiogenesis, extracellular matrix modeling, and regulation of inflammation and, hence, contribute to both the function and dysfunction of AT (Crewe et al., 2017).

Healthy white AT is a dynamic and flexible organ that stores fat when in excess and provides energy when needed. After a meal, insulin is secreted from pancreatic β-cells in response to increased levels of glucose, amino acids, and incretins in the blood (Wilcox, 2005). Insulin signaling in adipocytes through the insulin receptor increases glucose and fatty acid (FA) uptake into the adipocytes (Arner, 2005). Insulin suppresses lipolysis by increasing the activity of phosphodiesterase 3B (PDE3B), leading to decreased cAMP-mediated activation of protein kinase A (PKA), and, consequently, reduced phosphorylation of hormone-sensitive lipase (HSL) and perilipin (PLIN). This ultimately promotes lipogenesis, resulting in triglyceride storage and, therefore, adipocyte expansion (Zechner et al., 2009; Petersen and Shulman, 2018). In contrast, stressors such as exercise and starvation promote catecholamine secretion. Catecholamines act through β-adrenergic receptors in adipocytes, inducing an increase in cAMP levels, which activates PKA and ultimately enhances lipolysis (Arner, 2005). PKA activates lipolysis at several steps, including through PLIN and HSL phosphorylation. PLIN phosphorylates CGI-58, allowing it to bind to and fully activate ATGL, while phosphorylated HSL is directly involved in lipolysis (Zechner et al., 2009). When insulin levels are relatively low, AT releases FFAs into the bloodstream, making them available for muscle and other tissues when needed (Arner, 2005). Beta-oxidation accounts for 80% of the total energy-related oxidation in skeletal muscle at rest after an overnight fast, and this percentage increases further in response to prolonged fasting (Kelley and Simoneau, 1994). In skeletal muscle, FFAs are either stored as triglycerides in lipid droplets or metabolized via β-oxidation, providing energy for exercise and other energy-consuming processes (Kiens, 2006). Importantly, there is a continuous balance between the oxidation of glucose and fat during both rest and activity. Fat oxidation increases the levels of acetyl-CoA and inhibits glucose oxidation via the Randle cycle, whereas excessive glucose uptake increases malonyl-CoA levels, which inhibits fat oxidation (Hue and Taegtmeyer, 2009).

Metabolic flexibility, the ability to switch between fat and carbohydrate oxidation with varying energy requirements seems to be important for proper metabolic regulation (Goodpaster and Sparks, 2017). Metabolic flexibility is attenuated in obesity and more so in T2DM (Smith et al., 2018). In the resting state, obese people have a higher RER value when compared with lean individuals, meaning a larger fraction of the energy expended derives from carbohydrate oxidation, an effect that is even more pronounced in patients with T2DM (Goodpaster and Sparks, 2017). Furthermore, during insulin stimulation, obese individuals and patients with T2DM are not able to increase the RER value to the same extent as lean individuals (Kelley and Simoneau, 1994; Goodpaster and Sparks, 2017). However, during high-intensity exercise, obese individuals and patients with T2DM can increase the RER to values comparable to those observed in lean individuals (Kelley and Mandarino, 2000). The exact role of impaired metabolic flexibility remains unknown, and whether it is a consequence of insulin resistance or an early impairment that contributes to insulin resistance remains to be clarified.

Disrupted signaling along intracellular pathways responsible for lipid metabolism is one of the features of impaired metabolic flexibility in obesity (Boucher et al., 2014). As mentioned earlier, insulin primarily inhibits lipolysis while catecholamines primarily promote lipolysis. Insulin resistance in adipocytes is associated with impaired phosphorylation of insulin receptor substrate 1 (IRS-1) in obesity, which is further impaired in T2DM (Copps and White, 2012). The inability of insulin to inhibit lipolysis results in an increased efflux of FFAs into the bloodstream. The counter-regulatory pathway involving catecholamine-related HSL stimulation is also attenuated in obesity, rendering adipocytes insensitive to catecholamines (Arner, 2005). Thus, obesity reduces the flexibility to switch between lipolysis and lipogenesis, exacerbating a vicious cycle whereby an increase in lipolysis results in ectopic lipid deposition and further insulin resistance.

## Key Features of at Dysfunction

**FIGURE 2:**
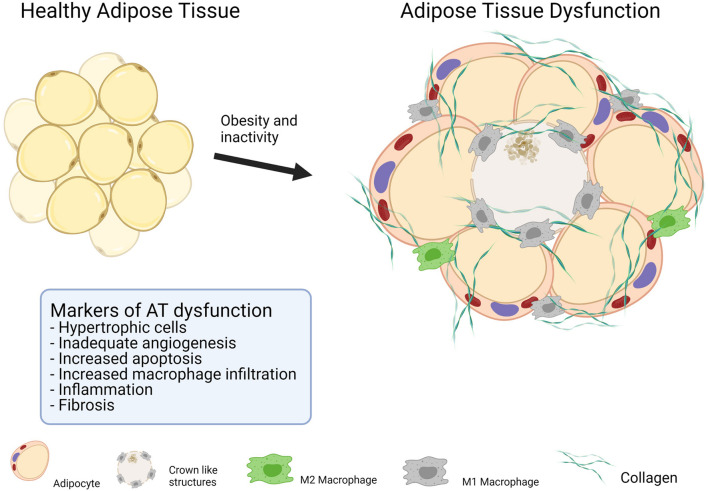
Makers of adipose tissue dysfunction.

The localization of fat depots is a strong predictor of metabolic diseases. Regardless of its localization, AT content and function differ markedly between obese and healthy lean people (Rydén et al., 2014), and is likely to be further altered in patients with T2DM compared with obese individuals with the same BMI (Camastra et al., 2017). Collectively, these changes are termed AT dysfunction (Crewe et al., 2017), which is characterized by an unhealthy AT expansion characterized by the presence of hypertrophic adipocytes, excessive accumulation of extracellular matrix (ECM) components, interstitial fibrosis, exaggerated pro-inflammatory macrophage infiltration, and dysregulated angiogenesis (Figure 2). Alterations in the ECM and impaired angiogenesis can be difficult to measure in human fat samples; however, the mRNA levels of proteins associated with the ECM and angiogenesis are often used as markers of these processes in AT dysfunction (Åkra et al., 2020). Low-grade inflammation, macrophage infiltration, and adipocyte cell size are frequently used to determine the degree of AT dysfunction (Longo et al., 2019).

AT dysfunction and the resulting impaired AT expandability cause local and systemic low-grade inflammation, fat overflow, and ectopic fat deposition, all of which are associated with insulin resistance (Crewe et al., 2017). Whether AT dysfunction is the cause or consequence of insulin resistance remains unknown. Furthermore, although the exact pathophysiological mechanism underlying NAFLD remains unknown, its development is associated with the insulin receptor, low-grade inflammation, and ectopic lipid deposition. Consequently, NAFLD is exacerbated with AT dysfunction (Cimini et al., 2017; Tarantino et al., 2019).

Hypertrophic adipocytes show a positive correlation with insulin resistance and the risk of CVD (Muir et al., 2016), while increases in macrophage infiltration and the numbers of apoptotic adipocytes are also seen in dysfunctional AT (Crewe et al., 2017). The exact sequence of events and pathways involved are still under debate. One of the most studied hypotheses postulates that relative hypoxia in AT mediates a cascade of events that lead to the increased apoptosis and low-grade inflammation seen in obesity (Trayhurn and Wood, 2004). Adipocytes increase in size as a result of obesity, with some becoming larger than 100 μm (Yin et al., 2009). At this distance, oxygen diffusion is compromised due to the limitation of the diffusion capacity. Hypoxia *per se* decreases insulin signaling and disrupts lipid metabolism in AT (Regazzetti et al., 2009; Yin et al., 2009). Furthermore, the cytokine hypoxia-inducible factor 1 alpha (HIF-1α) is upregulated in a hypoxic environment. HIF-1α is a multifunctional molecule best known for its function in promoting angiogenesis. However, in dysfunctional AT, HIF-1α fails to promote angiogenesis, inducing instead dysregulated ECM remodeling, resulting in fibrosis that disrupts proper angiogenesis, further exacerbating hypoxia in AT (Sun et al., 2013).

Although adipocytes undergo apoptosis under normal conditions, an increased rate is observed in obesity (Camastra et al., 2017). Adipocytes have a lifetime of approximately 8–10 years, indicating that ∼10% of these cells must be replaced annually (Spalding et al., 2008). Accordingly, a balance between apoptosis and adipogenesis is required for the maintenance of AT. Nevertheless, apoptotic cells attract macrophages. Evidence suggests that most macrophages in AT are in close proximity to apoptotic adipocytes (Murano et al., 2008). The finding that macrophage numbers are increased in AT of obese individuals is not surprising. Macrophages are the main cell type responsible for the increased low-grade inflammation seen in AT dysfunction. However, evidence suggests that it is CD8^+^ T lymphocytes that initiate the processes of inflammation in AT (Nishimura et al., 2009). Low-grade inflammation drives the secretion of a broad spectrum of cytokines, which further increase low-grade inflammation, resulting in a vicious cycle. Tumor necrosis factor-alpha (TNF-α), interleukin 1 beta (IL-1β), and monocyte chemoattractant protein-1 (MCP-1) are some of the best-characterized cytokines in AT. TNF-α induces an increase in the M1/M2 polarization ratio in macrophages. M1 macrophages enhance the pro-inflammatory response, whereas M2 macrophages have anti-inflammatory properties. Additionally, TNF-α inhibits both the insulin and the catecholamine signaling pathways in adipocytes, resulting in decreased metabolic flexibility (Langin and Arner, 2006; Guilherme et al., 2008). The blood levels of IL-1β are elevated in systemic rheumatic diseases, which is believed to mediate the inflammation seen in these conditions. The role of IL-1β in obesity-related low-grade inflammation is not completely understood, but it has been suggested that it induces β-cell damage in T2DM (Gabay et al., 2010; Eguchi and Manabe, 2013). MCP-1 is secreted from macrophages and is chemotactic for monocytes, inducing further macrophage infiltration into the AT (Sakurai et al., 2017).

Importantly, under these conditions, AT dysfunction disrupts fat metabolism in adipocytes at multiple sites. Notably, an increased resistance to insulin results in elevated lipolysis, resulting in elevated levels of circulating FFAs. In addition to insulin resistance, reduced sensitivity to catecholamines also decreases metabolic flexibility. Fat overflow, together with low-grade systemic inflammation, results in ectopic lipid deposition in the liver, muscle, and pancreatic β-cells. Lipid deposition in the liver and muscle directly increases insulin resistance (Snel et al., 2012), while in pancreatic β-cells it is believed to be lipotoxic, decreasing their ability to secrete insulin (Robertson et al., 2004). A combination of insulin resistance and decreased insulin secretion is expected to increase the risk for T2DM.

## Exercise and Metabolic Regulation

Physical activity is the cornerstone for improving metabolic health, and a lack of physical activity increases the risk of obesity and obesity-associated diseases such as T2DM, CVD, and NAFLD (Booth et al., 2017). For T2DM and CVD, a broad range of pharmaceuticals drugs are also available that can halt the progression of both diseases. In contrast, the pharmaceutical options for the management of NAFLD are limited (Tarantino et al., 2019). Because NAFLD has become the most common cause of chronic liver disease, it is important that therapeutic options for this condition are identified. Physical activity has proven to be an effective intervention for treating and preventing NAFLD (van der Windt et al., 2018; Wang et al., 2020). Exactly how exercise improves NAFLD is not completely understood (van der Windt et al., 2018); however, complex crosstalk between adipokines, hepatokines, and myokines has been suggested to play a central role in this improvement (de Oliveira Dos Santos et al., 2021).

The effect of exercise training on glucose tolerance and insulin sensitivity has been extensively researched (Sandvei et al., 2012; Langleite et al., 2016; Jelstad et al., 2019). The improved insulin sensitivity in skeletal muscle in response to exercise training can be in part explained by the upregulation of proteins involved in glucose metabolism and the insulin signaling pathway, including the insulin-sensitive glucose transporter 4 (GLUT4), hexokinase II, AKT2, glycogen synthase, and AMP-activated protein kinase (AMPK), as well as mitochondrial proteins (Wojtaszewski and Richter, 2006; Vind et al., 2011; Sylow et al., 2017). Although not as thoroughly investigated as skeletal muscle, it seems that at least some of these changes also happen in AT. In humans, insulin sensitivity in AT is accompanied by an increase in glucose transportation and glycolytic pathway (Riis et al., 2018). Furthermore, it has been shown that fat oxidation at rest increases in response to exercise, suggesting an improvement in AT/skeletal muscle axis (Calles-Escandón et al., 1996).

Sustained adherence to endurance exercise increases oxidative capacity and, consequently, the capacity to utilize energy. In many interventional exercise training studies, no significant reduction in body weight is seen either in obese or lean subjects, despite an improvement in metabolic regulation (Sandvei et al., 2012; Langleite et al., 2016). In exercise interventions, standardized work is normally performed at a specific workload or intensity for a given duration. For instance, in a study performed by our research group, aerobic training sessions involving ∼40 min of cycling at high intensity (75% *V*O_2*max*_) resulted in an energy expenditure of 600 kcal for untrained, lean, middle-aged males (Jelstad et al., 2019). Three weekly sessions each with a 600 kcal expenditure (a total of 1,800 kcal per week) correspond to the energy in 234 g of fat and 12 weeks of training intervention with such energy expenditure corresponds to the energy in 2.8 kg of fat. As previously mentioned, most of the energy used during high-intensity exercise is sourced from carbohydrates. Regardless of the source of the energy expended and the increased energy expenditure during exercise intervention, there must be a total negative energy balance to attain a reduction in body weight.

Exercise training can change body composition without changing total body weight by decreasing fat mass and increasing muscle mass (Langleite et al., 2016). In a 12-week training intervention consisting of 2 weekly sessions of HIIT and two sessions of resistance training, we observed a reduction in both subcutaneous and visceral fat deposits concomitant with an increase in muscle mass, resulting in no significant reduction in body weight (Langleite et al., 2016). From an energy perspective, 1 kg of fat contains 7,700 kcal, whereas muscle tissue is 70% water and the energy content in 1 kg of muscle is ∼1,000 kcal. The small increase in muscle mass has little effect on the REE because the metabolic rate in resting skeletal muscle is only ∼10–15 kcal/(kg⋅day^–1^) (Elia and Livesey, 1992). Although higher than the metabolic rate in AT ∼5 kcal/(kg⋅day^–1^), the increase accounts for only a small proportion of the whole-body energy expenditure. The brain, heart, kidney, liver, and gastrointestinal tract are the tissues that utilize the most energy at rest, and, for practical reasons, are not under the influence of the training (Elia and Livesey, 1992). Regardless of the small changes in REE, the AEE can increase in response to HIIT and SIT. As previously mentioned, these regimens increase muscle mass and oxidative phosphorylation (and *V*O_2*max*_), and, therefore, the ability to utilize more energy during a given time will be higher (Chrøis et al., 2020). There is evidence that HIIT promotes a greater improvement in mitochondrial oxidative capacity compared with MICT (MacInnis et al., 2017); however, to our knowledge, whether this can explain the increased fat loss has not been investigated.

Although energy expenditure is lower during SIT than during MICT, both regimens seem to undergo similar metabolic adaptations, such as increased *V*O_2*max*_ and a reduction in VAT, fasting glucose, and HbA1c (Burgomaster et al., 2008; Sandvei et al., 2012). The reasons for the similar improvements are not fully understood. Nonetheless, there is some evidence that a progressive increase in intensity results in a progressive increase in intracellular signaling in key metabolism-favorable pathways. High-intensity exercise activates both AMPK and peroxisome proliferator-activated receptor co-activator (PGC-1α) to a greater extent than low-intensity exercise in skeletal muscle (Wojtaszewski et al., 2000; Egan et al., 2010). AMPK is a pleiotropic protein with intracellular effects on lipid, glucose, and protein metabolism (Hardie et al., 2012), while PGC-1α is a potent driver of mitochondrial biogenesis and is also involved in glycogen metabolism (Wu et al., 1999; Wende et al., 2007; Kjøbsted et al., 2016). Furthermore, in a study comparing the metabolic effects of 10-fold higher energy expenditure in MICT compared with SIT, oxidative enzyme activity in muscle showed similar increases in both training regimens (Burgomaster et al., 2008). Whether this occurs in AT as well as in skeletal muscle remains to be investigated. Furthermore, the concentrations of catecholamines increase substantially during SIT, and anaerobic processes provide most of the energy during this workout. After 30 s “all-out,” the adrenaline concentration increases to high values (4 nM), more than double that in other types of exercise, resulting in an increased metabolic rate (Esbjörnsson-Liljedahl et al., 2002; Zouhal et al., 2008).

Among other functions, adrenaline increases glycogen breakdown. Increased intramuscular glycogen content is seen together with increased insulin sensitivity in response to several weeks of endurance exercise training (Vind et al., 2011). However, the glycogen store in muscles serves as the energy substrate during exercise, and an acute reduction in muscular glycogen content increases insulin sensitivity in the working muscle (Jensen et al., 1997, 2006, 2011). A single MICT session consisting of cycling for 60 min at 70% *V*O_2*max*_ reduced the glycogen amount by 50% in muscles (Pedersen et al., 2015). In comparison, a single SIT session stimulates glycogen breakdown and a single 30 s all-out cycling episode decreases the glycogen content in skeletal muscle by approximately 25% (Jacobs et al., 1982; Esbjörnsson-Liljedahl et al., 1999, 2002). Three bouts of 30 s all-out cycling with 20 min rest between sprints decreased glycogen content by approximately 50% (Esbjörnsson-Liljedahl et al., 2002). With only a total of 90 s of work, a SIT session results in the same reduction in glycogen content as 60 min of a MICT session.

## The Effect of Exercise on Abdominal Fat

The effect of exercise training on metabolic parameters, such as insulin sensitivity and fat loss, is unquestionably positive, even in the absence of a reduction in total body weight (Langleite et al., 2016). Exercise training can reduce abdominal visceral and subcutaneous fat mass even when there is no loss of total body weight. There is evidence to support that the relative reduction in VAT is greater than the reduction in SAT in response to exercise training in overweight people (Langleite et al., 2016). If exercise training is accompanied by a loss of total body weight, there is an even bigger relative reduction of visceral fat compared to the subcutaneous fat loss (Ross et al., 2000). If the speculative hypothesis that the amount of VAT increases when the capacity of SAT depots are exceeded, an improvement of AT function in response to exercise could also explain, why there is a relative larger loss of VAT.

While various meta-analyses have shown that MICT and HIIT leads to, at best, modest changes in body fat (Keating et al., 2017; Sultana et al., 2019), exercise intensity seems to play an important role in VAT modulation. A meta-analysis of the effect of exercise on VAT showed that high intensity exercise training induced a larger reduction of VAT compared to moderate and low intensity (Vissers et al., 2013), and moderate intensity exercise training (MICT) reduced VAT more than low intensity exercise training. This indicates that increasing intensity is associated with a greater reduction of VAT. The same study analyzed the effect of training volume (duration of sessions), and found no association between training volume and reduction of VAT or total fat mass. This is somewhat surprising, considering that fat is the predominant source of energy during low-moderate exercise. If the sympathetic nervous activation is greater with increasing exercise intensity, leading to greater VAT innervation, this may explain the increase in lipolysis in VAT and, thereby, also the reduction of VAT. Furthermore, a study has shown that insulin sensitivity, measured as increased glucose uptake using PET–CT combined with hyperinsulinemic clamp, increases in VAT in response to HIIT, but not moderate-intensity training (Honkala et al., 2020). There is evidence that exercise intensity is more important than duration for reducing VAT after a training period. This could in part explain why short-duration high-intensity exercise training can reduce fat, thereby obtaining good metabolic results. However, the associated molecular mechanisms remain unclear.

One of the mechanisms proposed to underlie the exercise-induced reduction of VAT involves signaling through IL-6. This cytokine is produced by a variety of cells, including adipocytes, lymphocytes, and macrophages. The plasma levels of IL-6 correlate well with systemic low-grade inflammation (Pedersen, 2017). Despite its positive correlation with disease, recent studies have suggested that IL-6 can also function as an anti-inflammatory cytokine (Pedersen, 2017)—an interesting duality. When IL-6 is secreted from contracting muscle tissue, it acts as an anti-inflammatory myokine, and is one of the first cytokines to be upregulated after exercise. An acute increase in IL-6 concentrations is believed to enhance whole-body lipolysis and β-oxidation (van Hall et al., 2003). Recently, blocking IL-6 with tocilizumab (an anti-IL-6 receptor antibody) was reported to abolish the reduction of visceral fat promoted by cycling exercise of unreported length and intensity three times a week. However, the average heart rate was 146 during exercise for participants between the ages of 40 and 45, which should be considered moderate intensity (Wedell-Neergaard et al., 2019). This highlights the importance of the crosstalk between muscle and AT, and also underlines the complexity of the effects associated with cytokines originating from different tissues and at different concentrations.

## The Effect of Exercise on at Dysfunction

**FIGURE 3:**
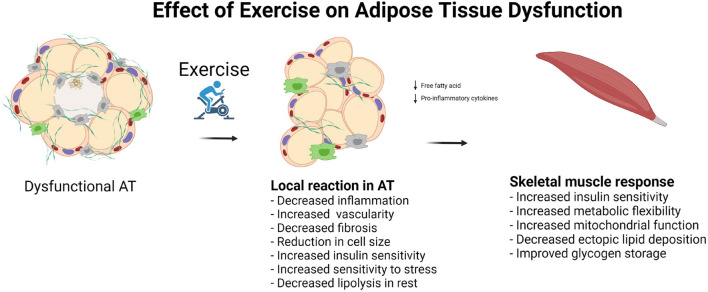
Effect of exercise on adipose tissue dysfunction.

In large cross-sectional population studies, moderate-to-high intensity physical activity is associated with reduced levels of circulating markers of low-grade inflammation, namely, IL-6, resistin, and leptin, and an increase in the concentration of the plasma anti-inflammatory marker adiponectin (Vella et al., 2017) (Figure 3). However, exercise interventions without weight loss have shown a limited ability to reduce the levels of known markers of low-grade inflammation (Allen et al., 2017; Kelly et al., 2017). In a study by Stinkens et al. (2018), which included obese men with and without T2DM, no changes in adipocyte cell size or the levels of markers of low-grade inflammation were detected after 12 weeks of exercise training consisting of two sessions of endurance exercise at 70% *V*O_2*max*_ for 30 min and one session of resistance training per week. The exercise in this study was of moderate intensity and relatively short duration. Furthermore, there was no loss of total body weight, although there was a 0.7-kg reduction in fat mass. Fat reduction was determined by DXA scan, and whether the abdominal fat was visceral or subcutaneous was not reported. Despite a lack of improvements in the concentrations of these markers of AT dysfunction, the authors found that insulin sensitivity was increased in skeletal muscle, but not in adipocytes or liver, as measured by hyperinsulinemic clamp (Stinkens et al., 2018). The participants had some degree of AT dysfunction related to obesity and T2DM; however, as the study did not include a lean, healthy control group, it is difficult to fully interpret the results.

Riis et al. (2018) investigated the molecular adaptations in AT in lean, healthy young male subjects [age 21 (18–24)] that underwent three sessions per week of endurance exercise of varying intensity for 10 weeks on a bicycle ergometer. They found that insulin sensitivity in AT, as measured using the AT insulin resistance index, was improved in the exercise group compared with that of the control group (Riis et al., 2018), concomitant with an increase in insulin receptor protein abundance as well as that of the downstream proteins involved in glucose oxidation in AT. Although the participants did not lose body weight, they displayed a significant reduction in fat mass. No changes in the levels of markers related to browning or beiging were reported in AT biopsies (Riis et al., 2018). Taken together, these observations suggest that exercise training can improve AT function, thereby contributing to improved metabolic flexibility. This study also emphasizes the positive effect of training on AT, even in young, lean, healthy individuals.

Exercise training alone has not been shown to reduce adipocyte size in obese individuals (Honkala et al., 2020). A meta-analysis of adipocyte cell size in overweight and obese individuals showed a linear relationship between the reduction of adipocyte cell size and the amount of weight lost (Murphy et al., 2017). In the same analysis, the authors compared the reduction of adipocyte cell size among different methods used to lose weight (bariatric surgery, dietary restriction, and exercise) and found no difference among the groups. This suggests that it may be necessary to reduce body weight in order to reduce adipocyte cell size (Figure 3).

In summary, regarding the effect of exercise training on AT, exercise *per se* does not reduce body weight. However, even without weight loss, exercise improves body composition by reducing fat mass and increasing muscle mass. Contracting skeletal muscle may play an important role in the reduction of VAT *via* the production and secretion of myokines such as IL-6. Insulin sensitivity in both adipocytes and skeletal muscle improves with exercise training. Despite the exercise-related improvement in AT function, the effect of exercise training on markers of AT dysfunction remains to be clarified. Evidence suggests that exercise *per se* does not reduce adipocyte cell size without weight loss; however, exercise training does, to some extent, reduce low-grade inflammation that is believed to evolve from AT dysfunction. Furthermore, exercise improves insulin sensitivity, glucose metabolism, and oxidative phosphorylation, all of which are impaired in dysfunctional AT.

## Conclusion/Perspectives

From an evolutionary perspective, AT stores energy for utilization in periods of low energy availability; however, a shortage of food does not often occur in the Western world, which has led to an increased prevalence of obesity and its related diseases. Obesity is not just an accumulation of fat in adipocytes but is also characterized by unfavorable lipid metabolism, AT dysfunction, ectopic lipid deposition, systemic low-grade inflammation, and insulin resistance. AT dysfunction may contribute to an unhealthy body fat distribution due to impaired fat storage in SAT, leading to an increase in VAT.

Nowadays, exercise training is often performed to lose weight and prevent lifestyle diseases. Losing weight through exercise can be an overwhelming task for untrained obese individuals, requiring a substantial amount of time. Nevertheless, even without inducing weight loss, exercise training reduces both total and visceral fat mass. HIIT and SIT aim to reduce the duration of exercise. Short-duration high-intensity exercise regimens are effective at reducing abdominal fat, indicating that they exert a positive effect on AT, which cannot be fully explained by the fat oxidized during the exercise bouts. A possible physiological mechanism explaining fat loss, in response to exercise at high intensities, is EPOC. The acute effects behind the increased EPOC does most likely not occur in the adipose tissue, but the energy can still be derived from AT. We argue that AT function is improved in response to increased energy utilization. Furthermore, AT dysfunction in obesity or T2DM is likely to be ameliorated in response to high-intensity exercise. However, the effect of exercise training on AT function and dysfunction, particularly with high intensity, remains to be thoroughly investigated.

## Author Contributions

KJK drafted the manuscript. TL-I, MHP, KH, and JJ commented on and read the final version. All authors contributed to the article and approved the submitted version.

## Conflict of Interest

The authors declare that the research was conducted in the absence of any commercial or financial relationships that could be construed as a potential conflict of interest.

## Publisher’s Note

All claims expressed in this article are solely those of the authors and do not necessarily represent those of their affiliated organizations, or those of the publisher, the editors and the reviewers. Any product that may be evaluated in this article, or claim that may be made by its manufacturer, is not guaranteed or endorsed by the publisher.

## References

[B1] Åkra S. Aksnes T. A. Flaa A. Eggesbø H. B. Opstad T. B. Njerve I. U. (2020). Markers of remodeling in subcutaneous adipose tissue are strongly associated with overweight and insulin sensitivity in healthy non-obese men. *Sci. Rep.* 10:14055. 10.1038/s41598-020-71109-4 32820223 PMC7441176

[B2] Allen N. G. Higham S. M. Mendham A. E. Kastelein T. E. Larsen P. S. Duffield R. (2017). The effect of high-intensity aerobic interval training on markers of systemic inflammation in sedentary populations. *Eur. J. Appl. Physiol.* 117 1249–1256. 10.1007/s00421-017-3613-1 28409397

[B3] Andersson Hall U. Edin F. Pedersen A. Madsen K. (2016). Whole-body fat oxidation increases more by prior exercise than overnight fasting in elite endurance athletes. *Appl. Physiol. Nutr. Metab.* 41 430–437. 10.1139/apnm-2015-0452 26988766

[B4] Arner P. (2005). Human fat cell lipolysis: biochemistry, regulation and clinical role. *Best Prac. Res. Clin. Endocrinol. Metab.* 19 471–482. 10.1016/j.beem.2005.07.004 16311212

[B5] Bækkerud F. H. Solberg F. Leinan I. M. Wisløff U. Karlsen T. Rognmo Ø (2016). Comparison of three popular exercise modalities on V^⋅^O2max in overweight and obese. *Med. Sci. Sports Exerc.* 48 491–498. 10.1249/MSS.0000000000000777 26440134

[B6] Bays H. E. Chapman R. H. Grandy S. (2007). The relationship of body mass index to diabetes mellitus, hypertension and dyslipidaemia: comparison of data from two national surveys. *Int. J. Clin. Pract.* 61 737–747. 10.1111/j.1742-1241.2007.01336.x 17493087 PMC1890993

[B7] Booth F. W. Roberts C. K. Thyfault J. P. Ruegsegger G. N. Toedebusch R. G. (2017). Role of inactivity in chronic diseases: evolutionary insight and pathophysiological mechanisms. *Physiol. Rev.* 97 1351–1402. 10.1152/physrev.00019.2016 28814614 PMC6347102

[B8] Boucher J. Kleinridders A. Kahn C. R. (2014). Insulin receptor signaling in normal and insulin-resistant states. *Cold Spring Harb. Perspect. Biol.* 6:a009191. 10.1101/cshperspect.a009191 24384568 PMC3941218

[B9] Bourin P. Bunnell B. A. Casteilla L. Dominici M. Katz A. J. March K. L. (2013). Stromal cells from the adipose tissue-derived stromal vascular fraction and culture expanded adipose tissue-derived stromal/stem cells: a joint statement of the International Federation for Adipose Therapeutics (IFATS) and Science and the International Society for Cellular Therapy (ISCT). *Cytotherapy* 15 641–648. 10.1016/j.jcyt.2013.02.006 23570660 PMC3979435

[B10] Burgomaster K. A. Howarth K. R. Phillips S. M. Rakobowchuk M. Macdonald M. J. McGee S. L. (2008). Similar metabolic adaptations during exercise after low volume sprint interval and traditional endurance training in humans. *J. Physiol. (Lond.).* 586 151–160. 10.1113/jphysiol.2007.142109 17991697 PMC2375551

[B11] Cahill G. F. (1970). Starvation in man. *N. Engl. J. Med.* 282 668–675. 10.1056/NEJM197003192821209 4915800

[B12] Calcagno M. Kahleova H. Alwarith J. Burgess N. N. Flores R. A. Busta M. L. (2019). The thermic effect of food: a review. *J. Am. Coll. Nutr.* 38 547–551. 10.1080/07315724.2018.1552544 31021710

[B13] Calles-Escandón J. Goran M. I. O’Connell M. Nair K. S. Danforth E. (1996). Exercise increases fat oxidation at rest unrelated to changes in energy balance or lipolysis. *Am. J. Physiol.* 270 E1009–E1014. 10.1152/ajpendo.1996.270.6.E1009 8764186

[B14] Camastra S. Vitali A. Anselmino M. Gastaldelli A. Bellini R. Berta R. (2017). Muscle and adipose tissue morphology, insulin sensitivity and beta-cell function in diabetic and nondiabetic obese patients: effects of bariatric surgery. *Sci. Rep.* 7:9007. 10.1038/s41598-017-08444-6 28827671 PMC5566429

[B15] Cannon B. Nedergaard J. (2011). Nonshivering thermogenesis and its adequate measurement in metabolic studies. *J. Exp. Biol.* 214 242–253. 10.1242/jeb.050989 21177944

[B16] Christ-Roberts C. Y. Pratipanawatr T. Pratipanawatr W. Berria R. Belfort R. Kashyap S. (2004). Exercise training increases glycogen synthase activity and GLUT4 expression but not insulin signaling in overweight nondiabetic and type 2 diabetic subjects. *Metabolism* 53 1233–1242. 10.1016/j.metabol.2004.03.022 15334390

[B17] Chrøis K. M. Dohlmann T. L. Søgaard D. Hansen C. V. Dela F. Helge J. W. (2020). Mitochondrial adaptations to high intensity interval training in older females and males. *Eur. J. Sport Sci.* 20 135–145. 10.1080/17461391.2019.1615556 31145037

[B18] Cimini F. A. Barchetta I. Carotti S. Bertoccini L. Baroni M. G. Vespasiani-Gentilucci U. (2017). Relationship between adipose tissue dysfunction, vitamin D deficiency and the pathogenesis of non-alcoholic fatty liver disease. *World J. Gastroenterol.* 23 3407–3417. 10.3748/wjg.v23.i19.3407 28596677 PMC5442077

[B19] Copps K. D. White M. F. (2012). Regulation of insulin sensitivity by serine/threonine phosphorylation of insulin receptor substrate proteins IRS1 and IRS2. *Diabetologia* 55 2565–2582. 10.1007/s00125-012-2644-8 22869320 PMC4011499

[B20] Crewe C. An Y. A. Scherer P. E. (2017). The ominous triad of adipose tissue dysfunction: inflammation, fibrosis, and impaired angiogenesis. *J. Clin. Invest.* 127 74–82. 10.1172/JCI88883 28045400 PMC5199684

[B21] de Oliveira Dos Santos A. R. de Oliveira Zanuso B. Miola V. F. B. Barbalho S. M. Santos Bueno P. C. Flato U. A. P. (2021). Adipokines, myokines, and hepatokines: crosstalk and metabolic repercussions. *Int. J. Mol. Sci.* 22:2639. 10.3390/ijms22052639 33807959 PMC7961600

[B22] Egan B. Carson B. P. Garcia-Roves P. M. Chibalin A. V. Sarsfield F. M. Barron N. (2010). Exercise intensity-dependent regulation of peroxisome proliferator-activated receptor coactivator-1 mRNA abundance is associated with differential activation of upstream signalling kinases in human skeletal muscle. *J. Physiol. (Lond.)* 588 1779–1790. 10.1113/jphysiol.2010.188011 20308248 PMC2887994

[B23] Eguchi K. Manabe I. (2013). Macrophages and islet inflammation in type 2 diabetes. *Diabetes Obes. Metab.* 15(Suppl. 3) 152–158. 10.1111/dom.12168 24003932

[B24] Elia M. Livesey G. (1992). Energy expenditure and fuel selection in biological systems: the theory and practice of calculations based on indirect calorimetry and tracer methods. *World Rev. Nutr. Diet.* 70 68–131. 10.1159/000421672 1292242

[B25] Esbjörnsson-Liljedahl M. Bodin K. Jansson E. (2002). Smaller muscle ATP reduction in women than in men by repeated bouts of sprint exercise. *J. Appl. Physiol.* 93 1075–1083. 10.1152/japplphysiol.00732.1999 12183505

[B26] Esbjörnsson-Liljedahl M. Sundberg C. J. Norman B. Jansson E. (1999). Metabolic response in type I and type II muscle fibers during a 30-s cycle sprint in men and women. *J. Appl. Physiol.* 87 1326–1332. 10.1152/jappl.1999.87.4.1326 10517759

[B27] Fealy C. E. Nieuwoudt S. Foucher J. A. Scelsi A. R. Malin S. K. Pagadala M. (2018). Functional high-intensity exercise training ameliorates insulin resistance and cardiometabolic risk factors in type 2 diabetes. *Exp. Physiol.* 103 985–994. 10.1113/EP086844 29766601 PMC6026040

[B28] Flatt J. P. (1995). Use and storage of carbohydrate and fat. *Am. J. Clin. Nutr.* 61 952S–959S. 10.1093/ajcn/61.4.952S 7900694

[B29] Fogelholm M. Malmberg J. Suni J. Santtila M. Kyröläinen H. Mäntysaari M. (2006). International physical activity questionnaire: validity against fitness. *Med. Sci. Sports Exerc.* 38 753–760. 10.1249/01.mss.0000194075.16960.20 16679993

[B30] Forouhi N. G. Wareham N. J. (2014). Epidemiology of diabetes. *Medicine (Abingdon)* 42 698–702. 10.1016/j.mpmed.2014.09.007 25568613 PMC4282306

[B31] Frayn K. N. (1983). Calculation of substrate oxidation rates in vivo from gaseous exchange. *J. Appl. Physiol. Respir. Environ. Exerc. Physiol.* 55 628–634. 10.1152/jappl.1983.55.2.628 6618956

[B32] Gabay C. Lamacchia C. Palmer G. (2010). IL-1 pathways in inflammation and human diseases. *Nat. Rev. Rheumatol.* 6 232–241. 10.1038/nrrheum.2010.4 20177398

[B33] Goodpaster B. H. Sparks L. M. (2017). Metabolic flexibility in health and disease. *Cell Metab.* 25 1027–1036. 10.1016/j.cmet.2017.04.015 28467922 PMC5513193

[B34] Granata C. Oliveira R. S. F. Little J. P. Renner K. Bishop D. J. (2016). Training intensity modulates changes in PGC-1α and p53 protein content and mitochondrial respiration, but not markers of mitochondrial content in human skeletal muscle. *FASEB J.* 30 959–970. 10.1096/fj.15-276907 26572168

[B35] Guilherme A. Virbasius J. V. Puri V. Czech M. P. (2008). Adipocyte dysfunctions linking obesity to insulin resistance and type 2 diabetes. *Nat. Rev. Mol. Cell Biol.* 9 367–377. 10.1038/nrm2391 18401346 PMC2886982

[B36] Hardie D. G. Ross F. A. Hawley S. A. (2012). AMPK: a nutrient and energy sensor that maintains energy homeostasis. *Nat. Rev. Mol. Cell Biol.* 13 251–262. 10.1038/nrm3311 22436748 PMC5726489

[B37] Henry C. J. K. (2005). Basal metabolic rate studies in humans: measurement and development of new equations. *Public Health Nutr.* 8 1133–1152. 10.1079/phn2005801 16277825

[B38] Heymsfield S. B. Thomas D. Martin C. K. Redman L. M. Strauss B. Bosy-Westphal A. (2012). Energy content of weight loss: kinetic features during voluntary caloric restriction. *Metabolism* 61 937–943. 10.1016/j.metabol.2011.11.012 22257646 PMC3810417

[B39] Honkala S. M. Motiani P. Kivelä R. Hemanthakumar K. A. Tolvanen E. Motiani K. K. (2020). Exercise training improves adipose tissue metabolism and vasculature regardless of baseline glucose tolerance and sex. *BMJ Open Diab. Res. Care* 8:e000830. 10.1136/bmjdrc-2019-000830 32816872 PMC7437884

[B40] Hue L. Taegtmeyer H. (2009). The randle cycle revisited: a new head for an old hat. *Am. J. Physiol. Endocrinol. Metab.* 297 E578–E591. 10.1152/ajpendo.00093.2009 19531645 PMC2739696

[B41] Jacobs I. Bar-Or O. Karlsson J. Dotan R. Tesch P. Kaiser P. (1982). Changes in muscle metabolites in females with 30-s exhaustive exercise. *Med. Sci. Sports Exerc.* 14 457–460. 10.1249/00005768-198206000-00009 7162392

[B42] Jelstad S. Ditta Valsdottir T. Johansen E. I. Jensen J. R. (2019). Eight sessions of endurance training decrease fasting glucose and improve glucose tolerance in middle-aged overweight males. *Arch. Physiol. Biochem.* 127 12–19. 10.1080/13813455.2018.1563189 30688111

[B43] Jensen J. Aslesen R. Ivy J. L. Brørs O. (1997). Role of glycogen concentration and epinephrine on glucose uptake in rat epitrochlearis muscle. *Am. J. Physiol.* 272 E649–E655. 10.1152/ajpendo.1997.272.4.E649 9142887

[B44] Jensen J. Jebens E. Brennesvik E. O. Ruzzin J. Soos M. A. Engebretsen E. M. L. (2006). Muscle glycogen inharmoniously regulates glycogen synthase activity, glucose uptake, and proximal insulin signaling. *Am. J. Physiol. Endocrinol. Metab.* 290 E154–E162. 10.1152/ajpendo.00330.2005 16118249

[B45] Jensen J. O’Rahilly S. (2017). AMPK is required for exercise to enhance insulin sensitivity in skeletal muscles. *Mol. Metab.* 6 315–316. 10.1016/j.molmet.2017.01.012 28377870 PMC5369262

[B46] Jensen J. Rustad P. I. Kolnes A. J. Lai Y.-C. (2011). The role of skeletal muscle glycogen breakdown for regulation of insulin sensitivity by exercise. *Front. Physiol.* 2:112. 10.3389/fphys.2011.00112 22232606 PMC3248697

[B47] Jetté M. Sidney K. Blümchen G. (1990). Metabolic equivalents (METS) in exercise testing, exercise prescription, and evaluation of functional capacity. *Clin. Cardiol.* 13 555–565. 10.1002/clc.4960130809 2204507

[B48] Karpe F. Pinnick K. E. (2015). Biology of upper-body and lower-body adipose tissue—link to whole-body phenotypes. *Nat. Rev. Endocrinol.* 11 90–100. 10.1038/nrendo.2014.185 25365922

[B49] Karstoft K. Brinkløv C. F. Thorsen I. K. Nielsen J. S. Ried-Larsen M. (2017). Resting metabolic rate does not change in response to different types of training in subjects with type 2 diabetes. *Front. Endocrinol. (Lausanne)* 8:132. 10.3389/fendo.2017.00132 28659869 PMC5468455

[B50] Keating S. E. Johnson N. A. Mielke G. I. Coombes J. S. (2017). A systematic review and meta-analysis of interval training versus moderate-intensity continuous training on body adiposity. *Obes. Rev.* 18 943–964. 10.1111/obr.12536 28513103

[B51] Kelley D. E. Mandarino L. J. (2000). Fuel selection in human skeletal muscle in insulin resistance: a reexamination. *Diabetes* 49 677–683. 10.2337/diabetes.49.5.677 10905472

[B52] Kelley D. E. Simoneau J. A. (1994). Impaired free fatty acid utilization by skeletal muscle in non-insulin-dependent diabetes mellitus. *J. Clin. Invest.* 94 2349–2356. 10.1172/JCI117600 7989591 PMC330064

[B53] Kelly B. M. Xenophontos S. King J. A. Nimmo M. A. (2017). An evaluation of low volume high-intensity intermittent training (HIIT) for health risk reduction in overweight and obese men. *BMC Obes.* 4:17. 10.1186/s40608-017-0151-7 28435687 PMC5395873

[B54] Kiens B. (2006). Skeletal muscle lipid metabolism in exercise and insulin resistance. *Physiol. Rev.* 86 205–243. 10.1152/physrev.00023.2004 16371598

[B55] Kjøbsted R. Wojtaszewski J. F. P. Treebak J. T. (2016). Role of AMP-activated protein kinase for regulating post-exercise insulin sensitivity. *Exp. Suppl.* 107 81–126. 10.1007/978-3-319-43589-3_5 27812978

[B56] Kopelman P. G. (2000). Obesity as a medical problem. *Nature* 404 635–643. 10.1038/35007508 10766250

[B57] Krogh A. Lindhard J. (1920). The changes in respiration at the transition from work to rest. *J. Physiol. (Lond.)* 53 431–439. 10.1113/jphysiol.1920.sp001889 16993427 PMC1405614

[B58] Kuo C.-H. Harris M. B. (2016). Abdominal fat reducing outcome of exercise training: fat burning or hydrocarbon source redistribution? *Can. J. Physiol. Pharmacol.* 94 695–698. 10.1139/cjpp-2015-0425 27152424

[B59] Langin D. Arner P. (2006). Importance of TNFalpha and neutral lipases in human adipose tissue lipolysis. *Trends Endocrinol. Metab.* 17 314–320. 10.1016/j.tem.2006.08.003 16938460

[B60] Langleite T. M. Jensen J. Norheim F. Gulseth H. L. Tangen D. S. Kolnes K. J. (2016). Insulin sensitivity, body composition and adipose depots following 12 w combined endurance and strength training in dysglycemic and normoglycemic sedentary men. *Arch. Physiol. Biochem.* 122 167–179. 10.1080/13813455.2016.1202985 27477619

[B61] Lenz M. Arts I. C. W. Peeters R. L. M. de Kok T. M. Ertaylan G. (2020). Adipose tissue in health and disease through the lens of its building blocks. *Sci. Rep.* 10:10433. 10.1038/s41598-020-67177-1 32591560 PMC7319996

[B62] Litleskare S. Enoksen E. Sandvei M. Støen L. Stensrud T. Johansen E. (2020). Sprint interval running and continuous running produce training specific adaptations, despite a similar improvement of aerobic endurance capacity-a randomized trial of healthy adults. *Int. J. Environ. Res. Public Health* 17:3865. 10.3390/ijerph17113865 32485945 PMC7312918

[B63] Longo M. Zatterale F. Naderi J. Parrillo L. Formisano P. Raciti G. A. (2019). Adipose tissue dysfunction as determinant of obesity-associated metabolic complications. *Int. J. Mol. Sci.* 20:2358. 10.3390/ijms20092358 31085992 PMC6539070

[B64] MacInnis M. J. Gibala M. J. (2017). Physiological adaptations to interval training and the role of exercise intensity. *J. Physiol.* 595 2915–2930. 10.1113/JP273196 27748956 PMC5407969

[B65] MacInnis M. J. Zacharewicz E. Martin B. J. Haikalis M. E. Skelly L. E. Tarnopolsky M. A. (2017). Superior mitochondrial adaptations in human skeletal muscle after interval compared to continuous single-leg cycling matched for total work. *J. Physiol.* 595 2955–2968. 10.1113/JP272570 27396440 PMC5407978

[B66] McCartney N. Spriet L. L. Heigenhauser G. J. Kowalchuk J. M. Sutton J. R. Jones N. L. (1986). Muscle power and metabolism in maximal intermittent exercise. *J. Appl. Physiol.* 60 1164–1169. 10.1152/jappl.1986.60.4.1164 3700299

[B67] Medbø J. I. Tabata I. (1989). Relative importance of aerobic and anaerobic energy release during short-lasting exhausting bicycle exercise. *J. Appl. Physiol.* 67 1881–1886. 10.1152/jappl.1989.67.5.1881 2600022

[B68] Meinild Lundby A.-K. Jacobs R. A. Gehrig S. de Leur J. Hauser M. Bonne T. C. (2018). Exercise training increases skeletal muscle mitochondrial volume density by enlargement of existing mitochondria and not de novo biogenesis. *Acta Physiol. (Oxf.).* 222 1–14. 10.1111/apha.12905 28580772

[B69] Moniz S. C. Islam H. Hazell T. J. (2020). Mechanistic and methodological perspectives on the impact of intense interval training on post-exercise metabolism. *Scand. J. Med. Sci. Sports* 30 638–651. 10.1111/sms.13610 31830334

[B70] Mooney S. J. Baecker A. Rundle A. G. (2013). Comparison of anthropometric and body composition measures as predictors of components of the metabolic syndrome in a clinical setting. *Obes. Res. Clin. Pract.* 7 e55–e66. 10.1016/j.orcp.2012.10.004 24331682

[B71] Muir L. A. Neeley C. K. Meyer K. A. Baker N. A. Brosius A. M. Washabaugh A. R. (2016). Adipose tissue fibrosis, hypertrophy, and hyperplasia: correlations with diabetes in human obesity: adipose tissue fibrosis in human obesity. *Obesity* 24 597–605. 10.1002/oby.21377 26916240 PMC4920141

[B72] Murano I. Barbatelli G. Parisani V. Latini C. Muzzonigro G. Castellucci M. (2008). Dead adipocytes, detected as crown-like structures, are prevalent in visceral fat depots of genetically obese mice. *J. Lipid Res.* 49 1562–1568. 10.1194/jlr.M800019-JLR200 18390487

[B73] Murphy J. Moullec G. Santosa S. (2017). Factors associated with adipocyte size reduction after weight loss interventions for overweight and obesity: a systematic review and meta-regression. *Metabolism* 67 31–40. 10.1016/j.metabol.2016.09.009 28081776

[B74] Ndahimana D. Kim E.-K. (2017). Measurement methods for physical activity and energy expenditure: a review. *Clin. Nutr. Res.* 6 68–80. 10.7762/cnr.2017.6.2.68 28503503 PMC5426207

[B75] Nguyen N. L. T. Randall J. Banfield B. W. Bartness T. J. (2014). Central sympathetic innervations to visceral and subcutaneous white adipose tissue. *Am. J. Physiol. Regul. Integr. Comp. Physiol.* 306 R375–R386. 10.1152/ajpregu.00552.2013 24452544 PMC3949107

[B76] Nishimura S. Manabe I. Nagasaki M. Eto K. Yamashita H. Ohsugi M. (2009). CD8+ effector T cells contribute to macrophage recruitment and adipose tissue inflammation in obesity. *Nat. Med.* 15 914–920. 10.1038/nm.1964 19633658

[B77] O’Rahilly S. (2016). Harveian Oration 2016: some observations on the causes and consequences of obesity. *Clin. Med. (Lond.).* 16 551–564. 10.7861/clinmedicine.16-6-551 27927821 PMC6297326

[B78] Park Y.-M. Myers M. Vieira-Potter V. J. (2014). Adipose tissue inflammation and metabolic dysfunction: role of exercise. *Mo. Med.* 111 65–72. 24645302 PMC6179510

[B79] Pedersen A. J. T. Hingst J. R. Friedrichsen M. Kristensen J. M. Højlund K. Wojtaszewski J. F. P. (2015). Dysregulation of muscle glycogen synthase in recovery from exercise in type 2 diabetes. *Diabetologia* 58 1569–1578. 10.1007/s00125-015-3582-z 25870023

[B80] Pedersen B. K. (2017). Anti-inflammatory effects of exercise: role in diabetes and cardiovascular disease. *Eur. J. Clin. Invest.* 47 600–611. 10.1111/eci.12781 28722106

[B81] Petersen M. C. Shulman G. I. (2018). Mechanisms of insulin action and insulin resistance. *Physiol. Rev.* 98 2133–2223. 10.1152/physrev.00063.2017 30067154 PMC6170977

[B82] Regazzetti C. Peraldi P. Grémeaux T. Najem-Lendom R. Ben-Sahra I. Cormont M. (2009). Hypoxia decreases insulin signaling pathways in adipocytes. *Diabetes* 58 95–103. 10.2337/db08-0457 18984735 PMC2606898

[B83] Riis S. Christensen B. Nellemann B. Møller A. B. Husted A. S. Pedersen S. B. (2018). Molecular adaptations in human subcutaneous adipose tissue after ten weeks of endurance exercise training in healthy males. *J. Appl. Physiol.* 126 569–577. 10.1152/japplphysiol.00989.2018 30571288

[B84] Robertson R. P. Harmon J. Tran P. O. T. Poitout V. (2004). β-Cell Glucose toxicity, lipotoxicity, and chronic oxidative stress in type 2 diabetes. *Diabetes* 53 S119–S124. 10.2337/diabetes.53.2007.S119 14749276

[B85] Romijn J. A. Coyle E. F. Sidossis L. S. Gastaldelli A. Horowitz J. F. Endert E. (1993). Regulation of endogenous fat and carbohydrate metabolism in relation to exercise intensity and duration. *Am. J. Physiol.* 265 E380–E391. 10.1152/ajpendo.1993.265.3.E380 8214047

[B86] Rosen E. D. Spiegelman B. M. (2014). What we talk about when we talk about fat. *Cell* 156 20–44. 10.1016/j.cell.2013.12.012 24439368 PMC3934003

[B87] Rosenkilde M. Reichkendler M. H. Auerbach P. Toräng S. Gram A. S. Ploug T. (2013). Appetite regulation in overweight, sedentary men after different amounts of endurance exercise: a randomized controlled trial. *J. Appl. Physiol.* 115 1599–1609. 10.1152/japplphysiol.00680.2013 24052035

[B88] Ross R. Dagnone D. Jones P. J. Smith H. Paddags A. Hudson R. (2000). Reduction in obesity and related comorbid conditions after diet-induced weight loss or exercise-induced weight loss in men. A randomized, controlled trial. *Ann. Intern. Med.* 133 92–103. 10.7326/0003-4819-133-2-200007180-00008 10896648

[B89] Rustad P. I. Sailer M. Cumming K. T. Jeppesen P. B. Kolnes K. J. Sollie O. (2016). Intake of protein plus carbohydrate during the first two hours after exhaustive cycling improves performance the following day. *PLoS One* 11:e0153229. 10.1371/journal.pone.0153229 27078151 PMC4831776

[B90] Rydén M. Andersson D. P. Bergström I. B. Arner P. (2014). Adipose tissue and metabolic alterations: regional differences in fat cell size and number matter, but differently: a cross-sectional study. *J. Clin. Endocrinol. Metab.* 99 E1870–E1876. 10.1210/jc.2014-1526 24937536

[B91] Sabag A. Way K. L. Sultana R. N. Keating S. E. Gerofi J. A. Chuter V. H. (2020). The effect of a novel low-volume aerobic exercise intervention on liver fat in type 2 diabetes: a randomized controlled trial. *Diabetes Care* 43 2371–2378. 10.2337/dc19-2523 32732374

[B92] Sakurai T. Ogasawara J. Shirato K. Izawa T. Oh-Ishi S. Ishibashi Y. (2017). Exercise training attenuates the dysregulated expression of adipokines and oxidative stress in white adipose tissue. *Oxid. Med. Cell Longev.* 2017:9410954. 10.1155/2017/9410954 28168013 PMC5266865

[B93] Sandvei M. Jeppesen P. B. Støen L. Litleskare S. Johansen E. Stensrud T. (2012). Sprint interval running increases insulin sensitivity in young healthy subjects. *Arch. Physiol. Biochem.* 118 139–147. 10.3109/13813455.2012.677454 22540332

[B94] Scheja L. Heeren J. (2019). The endocrine function of adipose tissues in health and cardiometabolic disease. *Nat. Rev. Endocrinol.* 15 507–524. 10.1038/s41574-019-0230-6 31296970

[B95] Smith R. L. Soeters M. R. Wüst R. C. I. Houtkooper R. H. (2018). Metabolic flexibility as an adaptation to energy resources and requirements in health and disease. *Endocr. Rev.* 39 489–517. 10.1210/er.2017-00211 29697773 PMC6093334

[B96] Snel M. Jonker J. T. Schoones J. Lamb H. de Roos A. Pijl H. (2012). Ectopic fat and insulin resistance: pathophysiology and effect of diet and lifestyle interventions. *Int. J. Endocrinol.* 2012:e983814. 10.1155/2012/983814 22675355 PMC3366269

[B97] Søgaard D. Lund M. T. Scheuer C. M. Dehlbaek M. S. Dideriksen S. G. Abildskov C. V. (2018). High-intensity interval training improves insulin sensitivity in older individuals. *Acta Physiol. (Oxf.).* 222:e13009. 10.1111/apha.13009 29197155

[B98] Sollie O. Jeppesen P. B. Tangen D. S. Jernerén F. Nellemann B. Valsdottir D. (2018). Protein intake in the early recovery period after exhaustive exercise improves performance the following day. *J. Appl. Physiol.* 125 1731–1742. 10.1152/japplphysiol.01132.2017 30212306

[B99] Spalding K. L. Arner E. Westermark P. O. Bernard S. Buchholz B. A. Bergmann O. (2008). Dynamics of fat cell turnover in humans. *Nature* 453 783–787. 10.1038/nature06902 18454136

[B100] Spriet L. L. Lindinger M. I. McKelvie R. S. Heigenhauser G. J. Jones N. L. (1989). Muscle glycogenolysis and H+ concentration during maximal intermittent cycling. *J. Appl. Physiol.* 66 8–13. 10.1152/jappl.1989.66.1.8 2917960

[B101] Stefan N. (2020). Causes, consequences, and treatment of metabolically unhealthy fat distribution. *Lancet Diabetes Endocrinol.* 8 616–627. 10.1016/S2213-8587(20)30110-8 32559477

[B102] Stefan N. Birkenfeld A. L. Schulze M. B. (2021). Global pandemics interconnected - obesity, impaired metabolic health and COVID-19. *Nat. Rev. Endocrinol.* 17 135–149. 10.1038/s41574-020-00462-1 33479538

[B103] Stewart W. K. Fleming L. W. (1973). Features of a successful therapeutic fast of 382 days’ duration. *Postgrad. Med. J.* 49 203–209. 10.1136/pgmj.49.569.203 4803438 PMC2495396

[B104] Stinkens R. Brouwers B. Jocken J. W. Blaak E. E. Teunissen-Beekman K. F. Hesselink M. K. (2018). Exercise training-induced effects on the abdominal subcutaneous adipose tissue phenotype in humans with obesity. *J. Appl. Physiol.* 125 1585–1593. 10.1152/japplphysiol.00496.2018 30212302

[B105] Stubbs R. J. O’Reilly L. M. Whybrow S. Fuller Z. Johnstone A. M. Livingstone M. B. E. (2014). Measuring the difference between actual and reported food intakes in the context of energy balance under laboratory conditions. *Br. J. Nutr.* 111 2032–2043. 10.1017/S0007114514000154 24635904

[B106] Sultana R. N. Sabag A. Keating S. E. Johnson N. A. (2019). The effect of low-volume high-intensity interval training on body composition and cardiorespiratory fitness: a systematic review and meta-analysis. *Sports Med.* 49 1687–1721. 10.1007/s40279-019-01167-w 31401727

[B107] Sun K. Tordjman J. Clément K. Scherer P. E. (2013). Fibrosis and adipose tissue dysfunction. *Cell Metab.* 18 470–477. 10.1016/j.cmet.2013.06.016 23954640 PMC3795900

[B108] Sylow L. Kleinert M. Richter E. A. Jensen T. E. (2017). Exercise-stimulated glucose uptake - regulation and implications for glycaemic control. *Nat. Rev. Endocrinol.* 13 133–148. 10.1038/nrendo.2016.162 27739515

[B109] Tarantino G. Citro V. Capone D. (2019). Nonalcoholic fatty liver disease: a challenge from mechanisms to therapy. *J. Clin. Med.* 9:E15. 10.3390/jcm9010015 31861591 PMC7019297

[B110] Tarnopolsky M. (2004). Protein requirements for endurance athletes. *Nutrition* 20 662–668. 10.1016/j.nut.2004.04.008 15212749

[B111] Tchernof A. Després J.-P. (2013). Pathophysiology of human visceral obesity: an update. *Physiol. Rev.* 93 359–404. 10.1152/physrev.00033.2011 23303913

[B112] Trapp E. G. Chisholm D. J. Freund J. Boutcher S. H. (2008). The effects of high-intensity intermittent exercise training on fat loss and fasting insulin levels of young women. *Int. J. Obes. (Lond.).* 32 684–691. 10.1038/sj.ijo.0803781 18197184

[B113] Trayhurn P. Wood I. S. (2004). Adipokines: inflammation and the pleiotropic role of white adipose tissue. *Br. J. Nutr.* 92 347–355. 10.1079/bjn20041213 15469638

[B114] Tucker W. J. Angadi S. S. Gaesser G. A. (2016). Excess postexercise oxygen consumption after high-intensity and sprint interval exercise, and continuous steady-state exercise. *J. Strength Cond. Res.* 30 3090–3097. 10.1519/JSC.0000000000001399 26950358

[B115] van der Windt D. J. Sud V. Zhang H. Tsung A. Huang H. (2018). The effects of physical exercise on fatty liver disease. *Gene Expr.* 18 89–101. 10.3727/105221617X15124844266408 29212576 PMC5954622

[B116] van Hall G. Steensberg A. Sacchetti M. Fischer C. Keller C. Schjerling P. (2003). Interleukin-6 stimulates lipolysis and fat oxidation in humans. *J. Clin. Endocrinol. Metab.* 88 3005–3010. 10.1210/jc.2002-021687 12843134

[B117] Van Pelt R. E. Evans E. M. Schechtman K. B. Ehsani A. A. Kohrt W. M. (2002). Contributions of total and regional fat mass to risk for cardiovascular disease in older women. *Am. J. Physiol. Endocrinol. Metab.* 282 E1023–E1028. 10.1152/ajpendo.00467.2001 11934666

[B118] Vella C. A. Allison M. A. Cushman M. Jenny N. S. Miles M. P. Larsen B. (2017). Physical activity and adiposity-related inflammation: the MESA. *Med. Sci. Sports Exerc.* 49 915–921. 10.1249/MSS.0000000000001179 27977529 PMC5392139

[B119] Vind B. F. Pehmøller C. Treebak J. T. Birk J. B. Hey-Mogensen M. Beck-Nielsen H. (2011). Impaired insulin-induced site-specific phosphorylation of TBC1 domain family, member 4 (TBC1D4) in skeletal muscle of type 2 diabetes patients is restored by endurance exercise-training. *Diabetologia* 54 157–167. 10.1007/s00125-010-1924-4 20938636

[B120] Virtue S. Vidal-Puig A. (2010). Adipose tissue expandability, lipotoxicity and the metabolic syndrome — an allostatic perspective. *Biochim. Biophys. Acta* 1801 338–349. 10.1016/j.bbalip.2009.12.006 20056169

[B121] Vissers D. Hens W. Taeymans J. Baeyens J.-P. Poortmans J. Van Gaal L. (2013). The effect of exercise on visceral adipose tissue in overweight adults: a systematic review and meta-analysis. *PLoS One* 8:e56415. 10.1371/journal.pone.0056415 23409182 PMC3568069

[B122] Wadden T. A. Webb V. L. Moran C. H. Bailer B. A. (2012). Lifestyle modification for obesity. *Circulation* 125 1157–1170. 10.1161/CIRCULATIONAHA.111.039453 22392863 PMC3313649

[B123] Wang S.-T. Zheng J. Peng H.-W. Cai X.-L. Pan X.-T. Li H.-Q. (2020). Physical activity intervention for non-diabetic patients with non-alcoholic fatty liver disease: a meta-analysis of randomized controlled trials. *BMC Gastroenterol.* 20:66. 10.1186/s12876-020-01204-3 32164541 PMC7066783

[B124] Wedell-Neergaard A.-S. Lang Lehrskov L. Christensen R. H. Legaard G. E. Dorph E. Larsen M. K. (2019). Exercise-induced changes in visceral adipose tissue mass are regulated by IL-6 signaling: a randomized controlled trial. *Cell Metab.* 29 844–855.e3. 10.1016/j.cmet.2018.12.007 30595477

[B125] Wells J. C. K. Fewtrell M. S. (2006). Measuring body composition. *Arch. Dis. Child.* 91 612–617. 10.1136/adc.2005.085522 16790722 PMC2082845

[B126] Wende A. R. Schaeffer P. J. Parker G. J. Zechner C. Han D.-H. Chen M. M. (2007). A role for the transcriptional coactivator PGC-1alpha in muscle refueling. *J. Biol. Chem.* 282 36642–36651. 10.1074/jbc.M707006200 17932032

[B127] Westerterp K. R. (2000). “Control of energy expenditure in humans,” in *Endotext*, eds Feingold K. R. Anawalt B. Boyce A. Chrousos G. de Herder W. W. Dungan K. (South Dartmouth, MA: MDText.com, Inc).

[B128] Westerterp K. R. (2004). Diet induced thermogenesis. *Nutr. Metab. (Lond.).* 1:5. 10.1186/1743-7075-1-5 15507147 PMC524030

[B129] Westerterp K. R. (2017). Doubly labelled water assessment of energy expenditure: principle, practice, and promise. *Eur. J. Appl. Physiol.* 117 1277–1285. 10.1007/s00421-017-3641-x 28508113 PMC5486561

[B130] Wilcox G. (2005). Insulin and insulin resistance. *Clin. Biochem. Rev.* 26 19–39. 16278749 PMC1204764

[B131] Winding K. M. Munch G. W. Iepsen U. W. Van Hall G. Pedersen B. K. Mortensen S. P. (2018). The effect on glycaemic control of low-volume high-intensity interval training versus endurance training in individuals with type 2 diabetes. *Diabetes Obes. Metab.* 20 1131–1139. 10.1111/dom.13198 29272072

[B132] Wojtaszewski J. F. P. Nielsen P. Hansen B. F. Richter E. A. Kiens B. (2000). Isoform-specific and exercise intensity-dependent activation of 5’-AMP-activated protein kinase in human skeletal muscle. *J. Physiol.* 528 221–226. 10.1111/j.1469-7793.2000.t01-1-00221.x 11018120 PMC2270117

[B133] Wojtaszewski J. F. P. Richter E. A. (2006). Effects of acute exercise and training on insulin action and sensitivity: focus on molecular mechanisms in muscle. *Essays Biochem.* 42 31–46. 10.1042/bse0420031 17144878

[B134] Wu Z. Puigserver P. Andersson U. Zhang C. Adelmant G. Mootha V. (1999). Mechanisms controlling mitochondrial biogenesis and respiration through the thermogenic coactivator PGC-1. *Cell* 98 115–124. 10.1016/S0092-8674(00)80611-X 10412986

[B135] Yin J. Gao Z. He Q. Zhou D. Guo Z. Ye J. (2009). Role of hypoxia in obesity-induced disorders of glucose and lipid metabolism in adipose tissue. *Am. J. Physiol. Endocrinol. Metab.* 296 E333–E342. 10.1152/ajpendo.90760.2008 19066318 PMC2645021

[B136] Zechner R. Kienesberger P. C. Haemmerle G. Zimmermann R. Lass A. (2009). Adipose triglyceride lipase and the lipolytic catabolism of cellular fat stores. *J. Lipid Res.* 50 3–21. 10.1194/jlr.R800031-JLR200 18952573

[B137] Zouhal H. Jacob C. Delamarche P. Gratas-Delamarche A. (2008). Catecholamines and the effects of exercise, training and gender. *Sports Med.* 38 401–423. 10.2165/00007256-200838050-00004 18416594

